# Therapeutic Response of miR-145 Micelles on Patient-Derived Vascular Smooth Muscle Cells

**DOI:** 10.3389/fdgth.2022.836579

**Published:** 2022-06-15

**Authors:** Neil Patel, Deborah D. Chin, Gregory A. Magee, Eun Ji Chung

**Affiliations:** ^1^Department of Biomedical Engineering, University of Southern California, Los Angeles, CA, United States; ^2^Division of Vascular Surgery and Endovascular Therapy, Department of Surgery, Keck School of Medicine, University of Southern California, Los Angeles, CA, United States; ^3^Mork Family Department of Chemical Engineering and Materials Science, University of Southern California, Los Angeles, CA, United States; ^4^Department of Medicine, Eli and Edythe Broad Center for Regenerative Medicine and Stem Cell Research, Keck School of Medicine, University of Southern California, Los Angeles, CA, United States; ^5^Division of Nephrology and Hypertension, Department of Medicine, Keck School of Medicine, University of Southern California, Los Angeles, CA, United States; ^6^Department of Medicine, Norris Comprehensive Cancer Center, Keck School of Medicine, University of Southern California, Los Angeles, CA, United States

**Keywords:** nanomedicine, miR-145, personalized medicine, plaque, vascular smooth muscle cell

## Abstract

During atherosclerosis, vascular smooth muscle cells (VSMCs) undergo a phenotypic transition from a healthy contractile state into pathological phenotypes including a proliferative and migratory, synthetic phenotype and osteochondrogenic-like phenotype that exacerbate plaques. Thus, inhibiting the transition of healthy, quiescent VSMCs to atherogenic cell types has the potential to mitigate atherosclerosis. To that end, previously, we reported that delivery of microRNA-145 (miR-145, a potent gatekeeper of the contractile VSMC phenotype) using nanoparticle micelles limited atherosclerotic plaque growth in murine models of atherosclerosis. Building on this preclinical data and toward clinical application, in this study, we tested the therapeutic viability of miR-145 micelles on patient-derived VSMCs and evaluated their effects based on disease severity. We collected vascular tissues from 11 patients with healthy, moderate, or severe stages of atherosclerosis that were discarded following vascular surgery or organ transplant, and isolated VSMCs from these tissues. We found that with increasing disease severity, patient-derived VSMCs had decreasing levels of contractile markers (miR-145, ACTA2, MYH11) and increasing levels of synthetic markers (KLF4, KLF5, and ELK1). Treatment with miR-145 micelles showed that an increase in disease severity correlated with a more robust response to therapy in VSMCs. Notably, miR-145 micelle therapy rescued contractile marker expression to baseline contractile levels in VSMCs derived from the most severely diseased tissues. As such, we demonstrate the use of miR-145 micelles across different stages of atherosclerosis disease and present further evidence of the translatability of miR-145 micelle treatment for atherosclerosis.

## Introduction

Heart disease and atherosclerosis continues to be the number one cause of death worldwide since 1969, despite significant advances in diagnosis and treatment (1). The most common drug used to prevent atherosclerosis focuses on reducing low density lipoprotein (LDL) cholesterol serum levels *via* statin therapy. Statin treatment however is marred by intolerance due to side effects as well as sub-optimal response in patients with secondary risk factors such as familial hyperlipidemia (2, 3). Thus, developing new therapies that are effective for the diverse patient population with atherosclerosis is needed.

Vascular smooth muscle cells (VSMCs) are central to plaque development (4). In early atherosclerosis, LDL cholesterol accumulates in the arterial intima, causing endothelial cell activation and monocyte infiltration. Macrophages and lipid-laden foam cells collect at these sites to form early lesions. Consequently, VSMCs dedifferentiate from a healthy contractile phenotype, which regulate vessel contraction, into a synthetic proliferative phenotype. Synthetic VSMCs have been reported to migrate from the media into lesions to secrete plaque-stabilizing collagen to protect plaques from rupture (5). However, VSMCs also dedifferentiate and transdifferentiate into alternative phenotypes including macrophage-like VSMCs that promote inflammation, foam cell-like VSMCs that contribute to the necrotic core, and osteochondrogenic-like VSMCs that secrete calcium minerals and advance the formation of rupture-prone plaques (6–9). Thus, targeting VSMC phenotype and transdifferentiation may be a strategy to combat atherosclerosis.

To that end, previously, we developed a novel therapeutic nanoparticle for the delivery of miR-145 micelles to VSMCs in atherosclerosis (10). miR-145 regulates the contractile VSMC phenotype and inhibits transcription factors associated with synthetic VSMCs such as Kruppel-like factor-4 (KLF4) and KLF5, and downregulation of miR-145 has been correlated with increasing atherosclerosis severity in patients and animal models of atherosclerosis (11, 12). miR-145 was incorporated into peptide amphiphile micelles as the micelle nanoparticle platform is ideally suited for delivering nucleic acids *in vivo* as it prevents nucleic acid degradation by nucleases and limits off target accumulation (10). Importantly, peptide amphiphile micelles are biocompatible as they are synthesized from biodegradable materials that are able to be cleared from the body even, after multiple doses as required in chronic diseases such as atherosclerosis, thereby limiting toxicity (13). Additionally, peptide amphiphile micelles have previously been shown to be effective in the treatment of atherosclerosis (13, 14) and we reported miR-145 micelles limited atherosclerotic plaque growth in both early- and late-stage murine models of atherosclerosis.

To continue to validate the translational capabilities of miR-145 micelles, herein, we collected and characterized atherosclerotic and vascular tissues from 11 patient donors and isolated and cultured VSMCs *in vitro*. We then characterized the effects of miR-145 micelles across patient-derived VSMCs and assessed the feasibility of miR-145 micelles in mitigating disease-propagating phenotypes. We show that miR-145 micelles show therapeutic efficacy in all samples, although the extent of therapeutic response varied based on disease severity. Notably, miR-145 micelles increased expression of contractile VSMC markers to levels similar to that of healthy VSMCs in cells derived from severely diseased tissue (sev-VSMCs). Taken together, we demonstrate the efficacy and mechanism of miR-145 micelles against atherosclerosis in patient-derived tissues and present further support for their clinical utility.

## Materials and Methods

### Synthesis of Peptide Amphiphiles

MCP-1 peptides were conjugated onto DSPE-PEG(2000)-maleimide using a previously reported protocol (10, 13–18). Briefly, 0.25 mmol MCP-1 peptide [YNFTNRKISVQRLASYRRITSSK] was synthesized with Wang resin *via* standard Fmoc-mediated, solid phase peptide synthesis methods on an automated peptide synthesizer (PS3, Protein Technologies, Tucson, AZ). To cleave the peptide from the resin, the resins was reacted with a solution of 94:2:5:2:5:1 vol% trifluoroacetic acid:1,2-ethanedithiol:water:triisopropylsilane for 4 h. Peptides were precipitated with ice-cold diethyl ether and washed twice. The crude peptides were dissolved in milliQ (MQ) water and lyophilized for storage until further use.

MCP-1 peptide was purified on a reverse-phase high performance liquid chromatography system (HPLC, Prominence, Shimadzu, Columbia, MD) with a gradient mobile phase consisting of (A) 0.1% (v/v) formic acid in water and (B) 0.1% (v/v) formic acid in acetonitrile at a column temperature of 55°C. The *m*/*z* was characterized by MALDI-TOF/TOF (Bruker, Billerica, MA, USA). The expected *m*/*z* peak is [M + H]^+^ = 2,890.

The purified peptide was conjugated to DSPE-PEG(2000)-maleimide in pH 7.4 buffered water for several days. The resulting product was purified using a C4 Jupiter column (250 × 30 mm ID, 5 μm, Phenomenex, Torrance, CA) as described above. The expected *m*/*z* peak for the peptide amphiphiles are [M + H]^+^ = 5,830.

### Synthesis of DSPE-PEG(2000)-miR-145 Mimics

miR-145 containing the sense sequence, 5′-GUCCAGUUUUCCCAGGAAUCCCU-3′, was custom ordered from IDT (Coralville, IA), and modified with a thiol group on the 5′ end of the sense (functional) strand for covalent conjugation to the micelle lipid tail. miR-145-SH (MW = 14,490 g/mol, 117.5 nmol, 1.70 mg) was added to DEPC-treated water to make 0.1 mM miR-145 solution. TCEP was added to the miR-145 solution and stirred in the dark at room temperature for 4 h at 1,600 rpm. Thiolated miR-145 was conjugated to DSPE-PEG(2000)-maleimide (Avanti Polar Lipids, Alabaster, AL) *via* a thioether bond by adding a 10% molar excess of lipid to reduced thiolated miR in DEPC-treated water. The resulting products were characterized using MALDI. The expected *m*/*z* peaks for DSPE-PEG(2000)-miR-145 was [M + H]^+^ = 17,047.

### Construction and Characterization of miR-Containing Micelles

miR-145 micelles were self-assembled by first dissolving MCP-1 peptide amphiphiles and DSPE-PEG(2000)-methoxy (49:50 mol ratio) in methanol. Methanol was completely evaporated under nitrogen and further vacuum dried overnight. The resulting film was hydrated with water, DSPE-PEG2000-miR-145 (1 mol%) in nuclease-free water added to the hydrated film, and the complete solution incubated at 60°C for 30 min. After incubation, the micelle solution was cooled to room temperature prior to use.

### Isolation of VSMCs From Patient Tissue

Discarded diseased and healthy patient tissues were collected from vascular operations at the Keck Hospital of USC and LAC + USC Medical Center. Specifically, discarded tissues included sections of the aorta, and the iliac, tibial, and carotid arteries. A total of 11 tissues from different patients were obtained and used for the studies. The use of human tissue was approved by the Institutional Review Board (IRB #HS-18-00638, BUA-18-0031) and Institutional Biosafety Committee (IBC #BUA-16-00057) at the University of Southern California. All tissues were obtained with informed consent.

Tissue samples were stored in Hank's balanced salt solution (HBSS) prior to VSMC harvest. Cells were isolated by washing the tissue three times with HBSS, removing the adventitial and endothelial layer, then cutting the tissue into 1–2 mm^2^ explants, and digesting with an enzyme cocktail consisting of 0.25% collagenase type II and 0.5% elastase type II for 16 h at 37°C. Isolated cells were then washed twice in media before culturing in Media 231 supplemented with Smooth Muscle Growth Supplements and 1% penicillin at 37°C. Images of cells were taken using brightfield of a fluorescence microscope (Leica DMI8, Wetzlar, Germany).

### Patient Tissue Histology

Portions of the patient tissue were saved for histological analysis. Tissues were flash frozen in optimal cutting temperature (OCT) and liquid nitrogen. Tissues were then sectioned in 10 μm thick slices and stained with hematoxylin and eosin (H&E). Calcium and cellular morphology in tissue were imaged and analyzed using a Leica DMI8 (Leica, Wetzlar, Germany) and ImageJ.

### Micelle Treatment of Patient VSMCs

Patient-derived VSMCs were seeded in 24-well plates at a density of 50,000 cells per well. Cells were then treated with miR-145 micelles (250 nM miR) or PBS for 4 h. miR-145 and mRNA expression of VSMC phenotypic markers were analyzed the next day *via* qRT-PCR.

### Gene Expression Analysis of VSMCs

RNA was isolated from VSMCs before or after miR-145 micelle treatment following the manufacturer's protocol using Qiagen miRNeasy mini kits. cDNA was synthesized using the RT^2^ First Strand Kit (Qiagen, Hilden, Germany) based on manufacturer's instructions. ACTA2, MYH11, MGP, KLF4, KLF5, ELK1, RUNX2 and miR-145 expressions were determined by real time-PCR using RT^2^ SYBR Green qPCR Mastermix (Qiagen, Hilden, Germany) on a CFX384 Real-Time PCR Detection System (Bio-Rad Laboratories, Hercules, CA). GAPDH or RNU6 was used as an internal loading control. The 2^−ΔΔCT^ method was used to quantify mRNA expression level.

### Statistical Analysis

Results were expressed as means ± standard deviation (SD). Statistical significance between two groups was determined using a two-tailed Student *t*-tests, while a one-way analysis of variance (ANOVA) was used to determine statistical significance between more than two groups. A *p* value < 0.05 was considered statistically significant. All statistical analyses were conducted using GraphPad Prism 8 (GraphPad Software, San Diego, CA).

## Results

### Patient-Derived Vascular Smooth Muscle Cell Characterization

**Figure 1:**
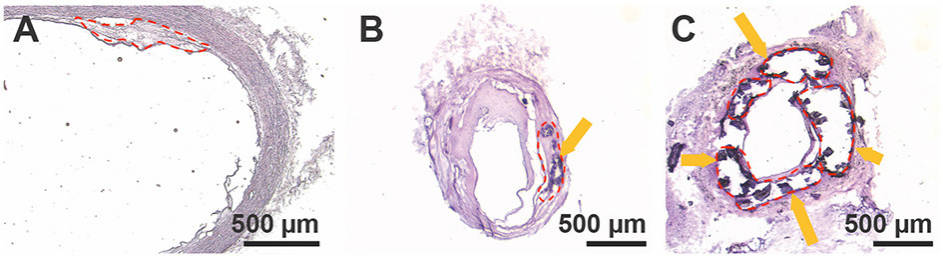
H&E stains of **(A)** healthy patient aortic tissue (lesion area outlined in red, 0.097 mm^2^), **(B)** moderately diseased patient tibial artery (lesion area 0.177 mm^2^), and **(C)** severely diseased patient tibial artery (lesion area 0.65 mm^2^). Dark purple stains and arrows indicate where bulk calcifications in the tissue are present.

Discarded healthy and atherosclerotic vascular tissues were obtained from organ donor tissues and patients undergoing open vascular surgery operations, respectively. Each sample was numbered and characterized by disease severity based on the extent of tissue calcification and luminal stenosis (e.g., none, moderate, severe) at the time of harvest by the operating surgeon (Table 1). Figure 1A shows a histological cross section of a healthy artery stained with H&E (sample 1) with little to no calcification and plaque development. Additionally, histological cross sections of a moderately and severely diseased artery (samples 6 and 8, respectively) show increasing plaque area, calcification, and occlusion of the vessel (Figures 1B,C) (19, 20).

**Table 1:** VSMCs isolated from vessels classified by disease severity based on the extent of calcification and plaque area.

**Sample ID**	**Disease severity**	**Disease**	**Sex**
**Healthy VSMCs**			
1	None	Unknown	Male
2	None	Unknown	Male
3	None	Unknown	Male
4	None	Unknown	Male
**Diseased VSMCs**			
5	Moderate	Carotid artery disease	Male
6	Moderate	Peripheral artery disease	Female
7	Moderate	Aortoiliac occlusive disease	Female
8	Severe	Peripheral artery disease	Female
9	Severe	Peripheral artery disease	Female
10	Severe	Peripheral artery disease	Male
11	Severe	Circumferential aortic calcification	Male

**Figure 2:**
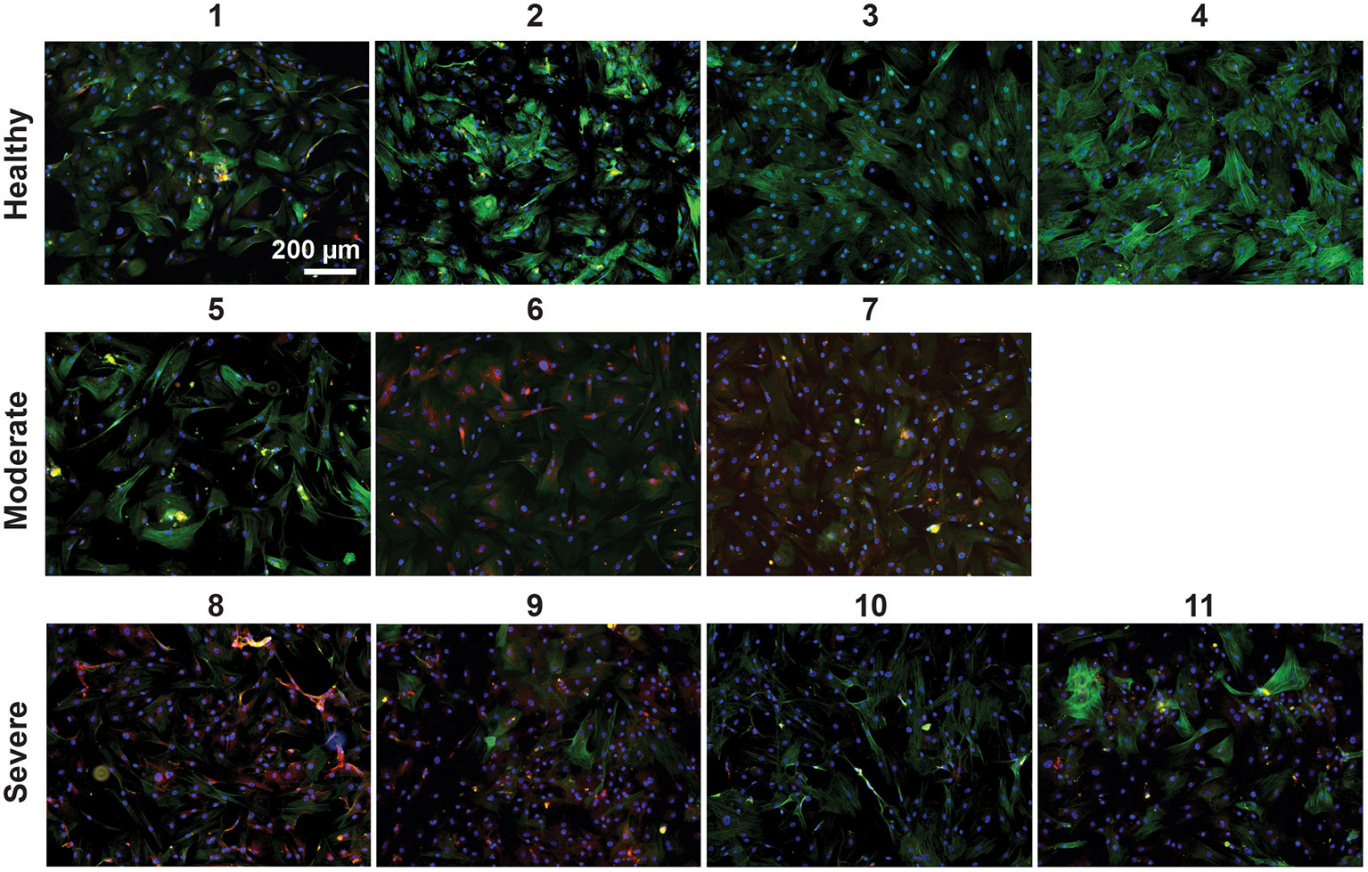
VSMCs isolated from healthy (1–4), moderately (5–7), and severely (8–11) diseased patient arteries were stained for the contractile marker, ACTA2 (green), the atherogenic inflammatory marker, CD68 (red), and nuclei with DAPI (blue). Healthy VSMCs show greater ACTA2 expression and sev-VSMCs show greater CD68 expression.

Next, we isolated VSMCs from all harvested arteries and performed immunocytochemical analyses. VSMCs derived from healthy tissue had upregulation of the contractile smooth muscle marker α-smooth muscle actin (ACTA2) compared to the moderately diseased tissue-derived VSMCs (mod-VSMCs) or the severely diseased tissue-derived VSMCs (sev-VSMCs; Figure 2, Supplementary Figure S2). Conversely, the proinflammatory atherogenic marker CD68 was upregulated in the mod-VSMCs and sev-VSMCs compared to the healthy VSMCs (hel-VSMCs). Interestingly, there were additional differences in the morphologies and characteristics of patient-derived VSMCs. For example, some hel-VSMCs (samples 1 and 2) took longer to adhere to tissue culture plates compared to sev-VSMCs upon seeding, although, by 24 h, all cells showed adherence and there were no significant differences in morphology (Supplementary Figure S1).

**Figure 3:**
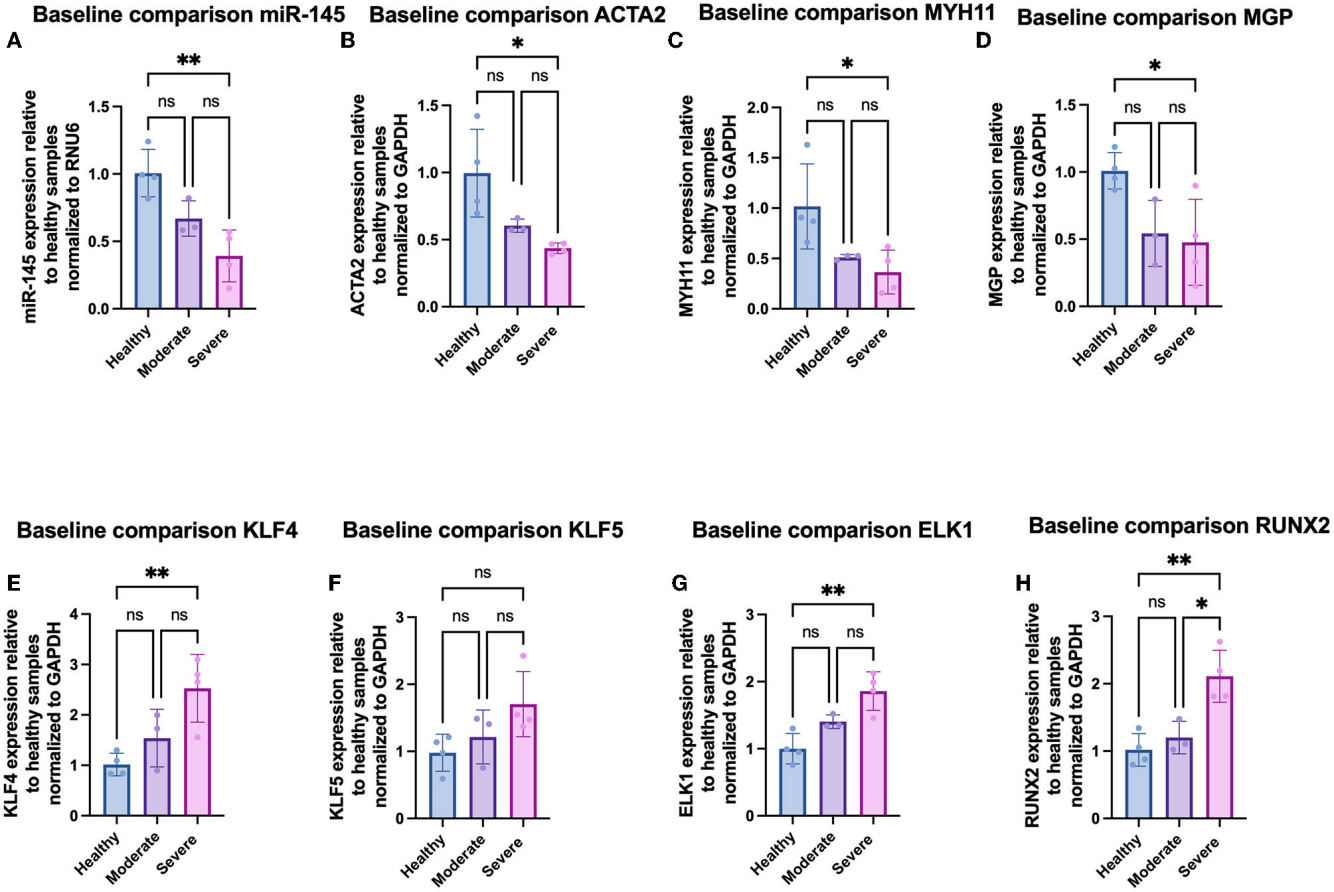
Gene expression of **(A)** miR-145, **(B)** ACTA2, **(C)** MYH11, **(D)** MGP, **(E)** KLF4, **(F)** KLF5, **(G)** ELK1, and **(H)** RUNX2 in VSMCs derived from varying disease severity. *p < 0.05, **p < 0.01.

We further analyzed phenotypic differences in VSMCs by looking at mRNA expression of a panel of contractile [ACTA2, smooth muscle myosin heavy chain-11 (MYH11), and matrix gla protein (MGP)], atherogenic [KLF4, KLF5, and ETS-like 1 protein (ELK1)], and osteochondrogenic [runt-related transcription factor 2 (RUNX2)] phenotypic markers *via* qRT-PCR. In addition, we quantified the expression of miR-145 within the cells. Compared to hel-VSMCs, miR-145 expression for mod-VSMCs and sev-VSMCs was decreased by 66.7 ± 13.2% (*p* = 0.078) and 42.9 ± 23.5% (*p* = 0.0043), respectively (21, 22). In addition, sev-VSMCs showed significantly decreased baseline expression of contractile genes ACTA2 (43.6 ± 3.9%), MYH11 (36.0 ± 27.6%), and MGP (44.4 ± 26.5%) compared to hel-VSMCs (Figures 3B–D) (6, 21, 23). While not statistically significant, mod-VSMCs showed decreased contractile gene expression: ACTA2 (60.3 ± 4.9%, *p* = 0.082), MYH11 (51.4 ± 2.7%, *p* = 0.08), MGP (54.3 ± 24.6%, *p* = 0.056). For synthetic phenotype markers, sev-VSMCs showed significantly increased KLF4 (261.5 ± 71.6%), KLF5 (175.7 ± 58.8%), ELK1 (185.9 ± 28.8%), and RUNX2 (214.4 ± 40.1%) expression compared to hel-VSMCs (Figures 3E–H) (6, 23–25). Similar trends were present for mod-VSMCs, but were not statistically significant (Figure 3).

**Figure 4:**
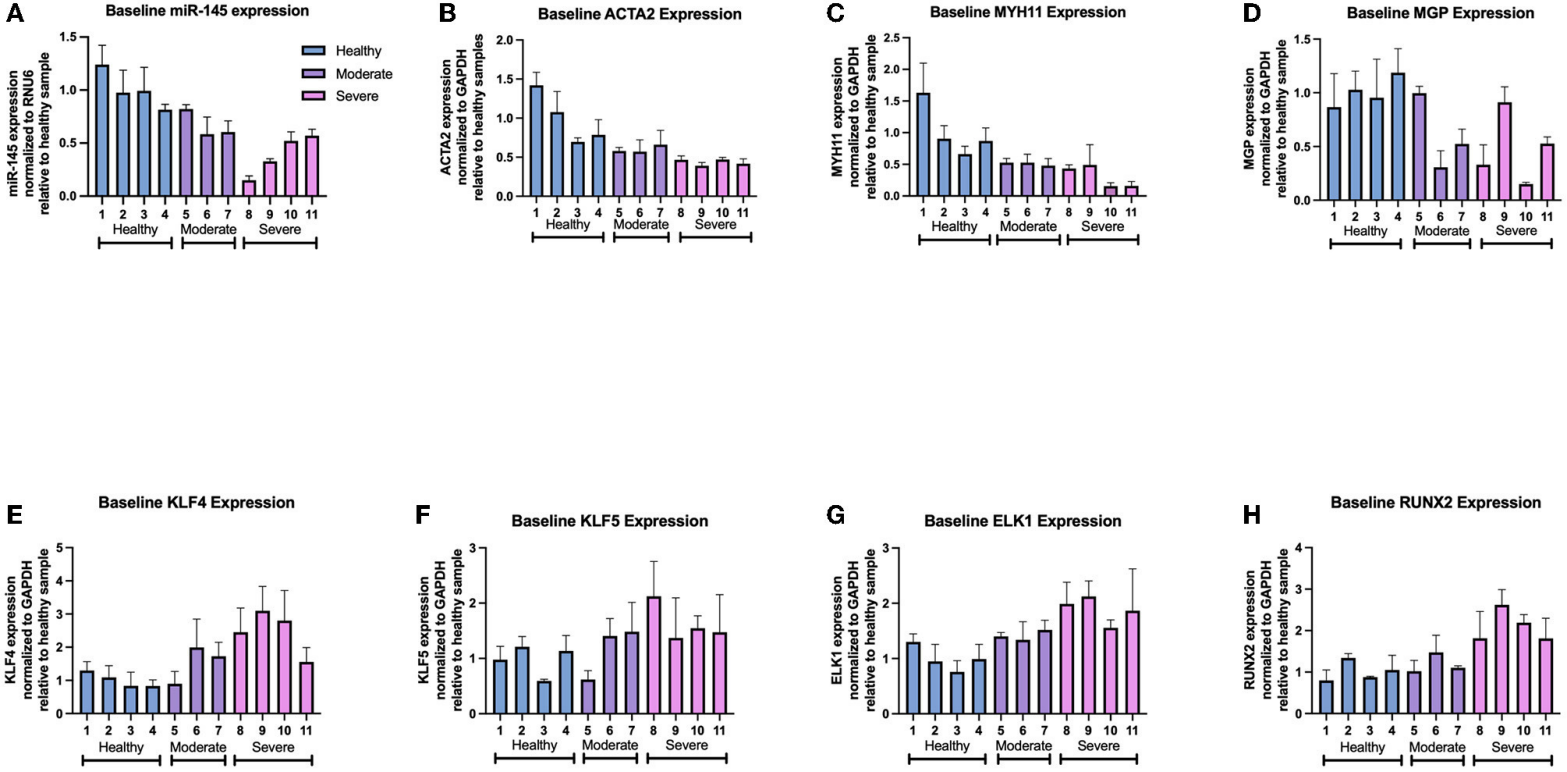
Gene expression of individual patient-derived VSMCs for **(A)** miR-145, **(B)** ACTA2, **(C)** MYH11, **(D)** MGP, **(E)** KLF4, **(F)** KLF5, **(G)** ELK1, and **(H)** RUNX2 levels.

Further analysis of individual samples from each group showed variations in gene expression between samples derived from arteries of similar disease severity. Within the moderately diseased group, sample 5 showed miR-145, MGP, KLF4, and KLF5 expression (82.1 ± 4.1%, 99.7 ± 6.3%, 89.7 ± 37.2%, 111.2 ± 19.6%) at levels similar to the healthy average VSMC baseline (Figures 4A–F). Within the severely diseased group, sample 8 had lower miR-145 expression relative to the other severely diseased samples (Figure 4A). Similarly, sample 11 within the severely diseased group had a baseline KLF4 expression (155.8 ± 43.2%) that is lower relative to other sev-VSMCs (Figure 4E). Of note, MGP expression varied within moderate and severe disease groups which is reflective of the current ambiguity with regards to MGP in the field of VSMC mediated-vascular calcification (Figure 4D) (25–28).

### VSMC Response to miR-145 Micelle Treatment

**Figure 5:**
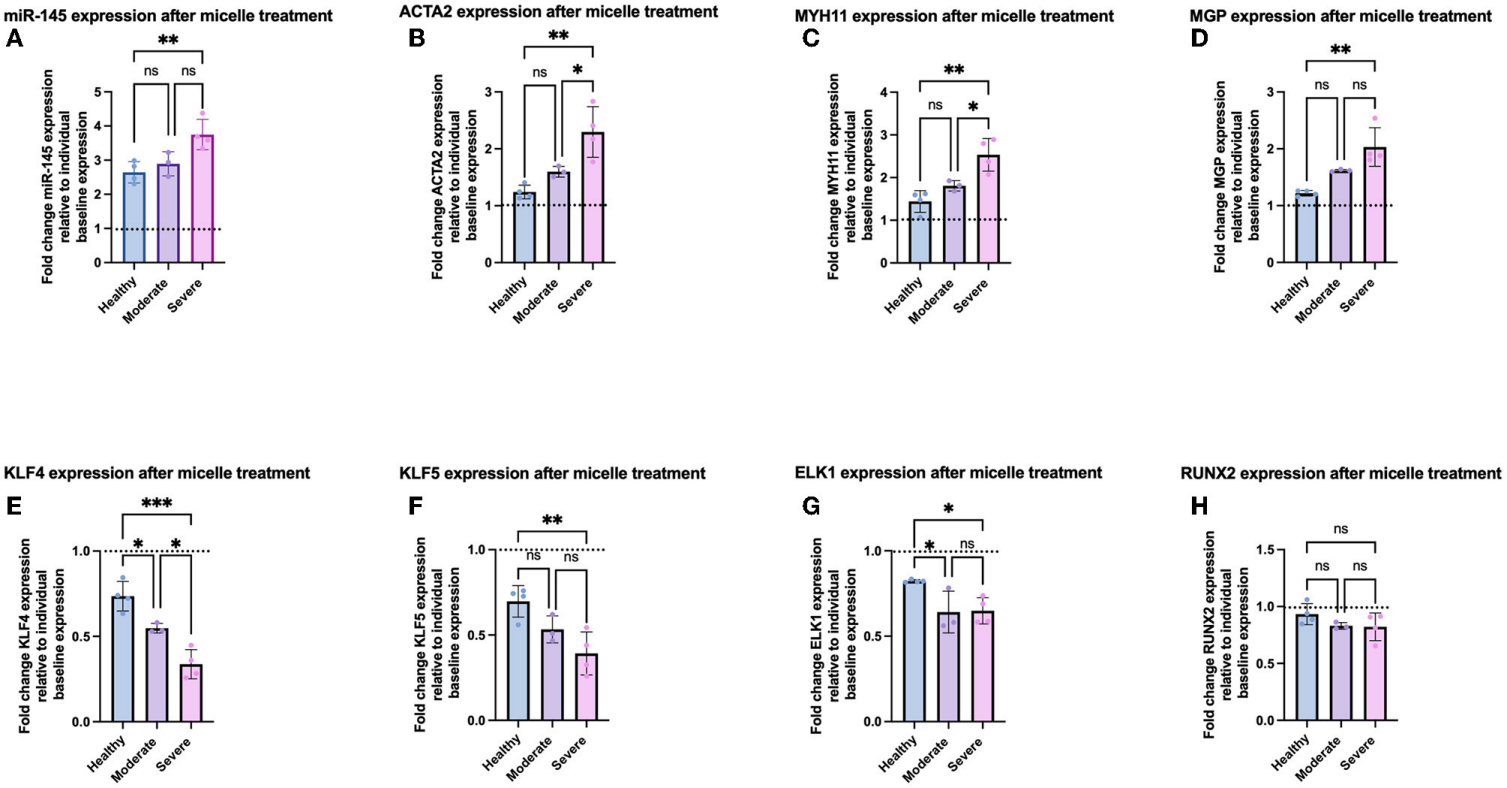
Gene expression of VSMCs after miR-145 micelle treatment based on disease severity. qRT-PCR analysis shows increased **(A)** miR-145 levels, **(B)** ACTA2, **(C)** MYH11, **(D**) MGP, and decreased **(E)** KLF4, **(F)** KLF5, **(G)** ELK1, and **(H)** RUNX2. Dotted line indicates individual baseline gene expression. *p < 0.05, **p < 0.01, and ***p < 0.001.

After characterization of baseline expression of VSMC phenotypic markers, we synthesized miR-145 micelles (Supplementary Figure S3) and investigated their effects on VSMC phenotype in each group. As mentioned earlier, miR-145 micelles have previously been shown to successfully enter VSMCs, release their miR cargo into the cytosol, and inhibit atherosclerosis progression in murine models (10). To test if miR-145 can exert therapeutic effects against VSMCs at varying disease severity, we incubated all patient-derived VSMCs with miR-145 micelles for 4 h and evaluated gene expression after 24 h *via* qRT-PCR (10, 12). miR-145 micelle therapy substantially elevated miR-145 levels for all VSMCs (hel-VSMCs: 264.4 ± 31.4%), with a more profound response in mod-VSMCs (296.0 ± 15.5%) and sev-VSMCs (371.2 ± 47.3%), relative to their pre-treated baseline levels (Figure 5A, Supplementary Figure S5A). Accordingly, contractile gene expression was significantly increased for both mod- and sev-VSMCs as follows: ACTA2 moderate (159.8 ± 9.3%) and severe (229.6 ± 44.4%), MYH11 moderate (184.1 ± 13.7%) and severe (253.3 ± 38.2%), and MGP moderate (155.1 ± 7.6%) and severe (185.7 ± 40.4%) relative to individual baseline values (Figures 5B–D, Supplementary Figures S4B–D). Conversely, synthetic VSMC markers in mod- and sev-VSMCs were significantly downregulated by miR-145 micelle treatment: KLF4 moderate (51.5 ± 8.4%) and severe (33.7 ± 8.6%), KLF5 moderate (53.3 ± 7.9%) and severe (39.2 ± 12.6%), and ELK1 moderate (64.0 ± 11.9%) and severe (63.3 ± 8.4%) relative to individual baseline values (Figures 5E–G, Supplementary Figures S4E–G) (29). For hel-VSMCs, miR-145 micelle treatment also led to statistically significant decreases in synthetic phenotype markers KLF4 (73.5 ± 8.6%), KLF5 (69.7 ± 9.3%), and ELK1 (82.3 ± 8.0%; Supplementary Figures S4E–G). However, this did not translate into significant increases in contractile markers (Supplementary Figures S4B–D). Notably, the osteochondrogenic marker, RUNX2, expression was not affected by miR-145 micelle treatment for all groups (Supplementary Figure S4H). This may be because upregulation of RUNX2 occurs independently of the myocardin signaling axis during the VSMC phenotype transition to an osteochondrogenic-like phenotype. Hence, while miR-145 increases myocardin expression by inhibiting KLF4, this does not lead to a direct decrease in RUNX2 expression (30).

Interestingly, the percent change in gene expression of contractile and synthetic VSMC markers relative to individual sample baselines after treatment with miR-145 micelles correlated with disease severity by group (healthy < moderate < severe disease). On average, hel-VSMCs miR-145 expression after micelle treatment increased by 264.4 ± 31.4% relative to the individual sample baselines (Figure 5A). This was correlated with increases in contractile gene expression [ACTA2 (122.8 ± 13.6%), MYH11 (144.0 ± 25.4%), and MGP (121.8 ± 4.8%)] and decreases in synthetic markers [KLF4 (73.5 ± 8.6%), KLF5 (69.7 ± 9.3%), and ELK1 (82.2 ± 1.8%); Figures 5B–G]. The percent change in gene expression was found to be greater upon micelle treatment for mod and sev-VSMCs compared to their baseline values vs. hel-VSMCs response; for mod-VSMCs: 296.0 ± 15.5% miR-145, 159.8 ± 9.3% ACTA2, 184.1 ± 13.6% MYH11, 161.8 ± 2.9% MGP, 54.8 ± 3.8% KLF4, 53.2 ± 7.9% KLF5, and 63.9 ± 11.9% ELK1 (Figures 5B–G). While increased, statistical significance between the delta in gene expression between hel- and mod-VSMCs occurred for only KLF4 and ELK1. Importantly, miR-145 micelle treatment rescued contractile markers in mod-VSMCs to hel-VSMC baseline levels (Supplementary Figure S5). For sev-VSMCs, miR-145 micelle treatment increased miR-145 and contractile markers gene expression [miR-145 (371.2 ± 47.3%), ACTA2 (229.6 ± 44.4%), MYH11 (253.3 ± 40.2), and MGP (203.2 ± 34.9%)] and decreased expression of synthetic markers [KLF4 (33.7 ± 8.6), KLF5 (39.2 ± 12.6%), ELK1 (64.3 ± 9.4%)] to the greatest extent when compared to the baseline sev-VSMC values (Figures 5B–G). Similar to mod-VSMCs, contractile markers were also restored to comparable healthy VSMC baseline levels (Supplementary Figure S5). The synthetic markers KLF4, KLF5, and ELK1 were also decreased after miR-145 micelle treatment in diseased VSMCs to a level close to healthy baseline values (Supplementary Figure S5).

**Figure 6:**
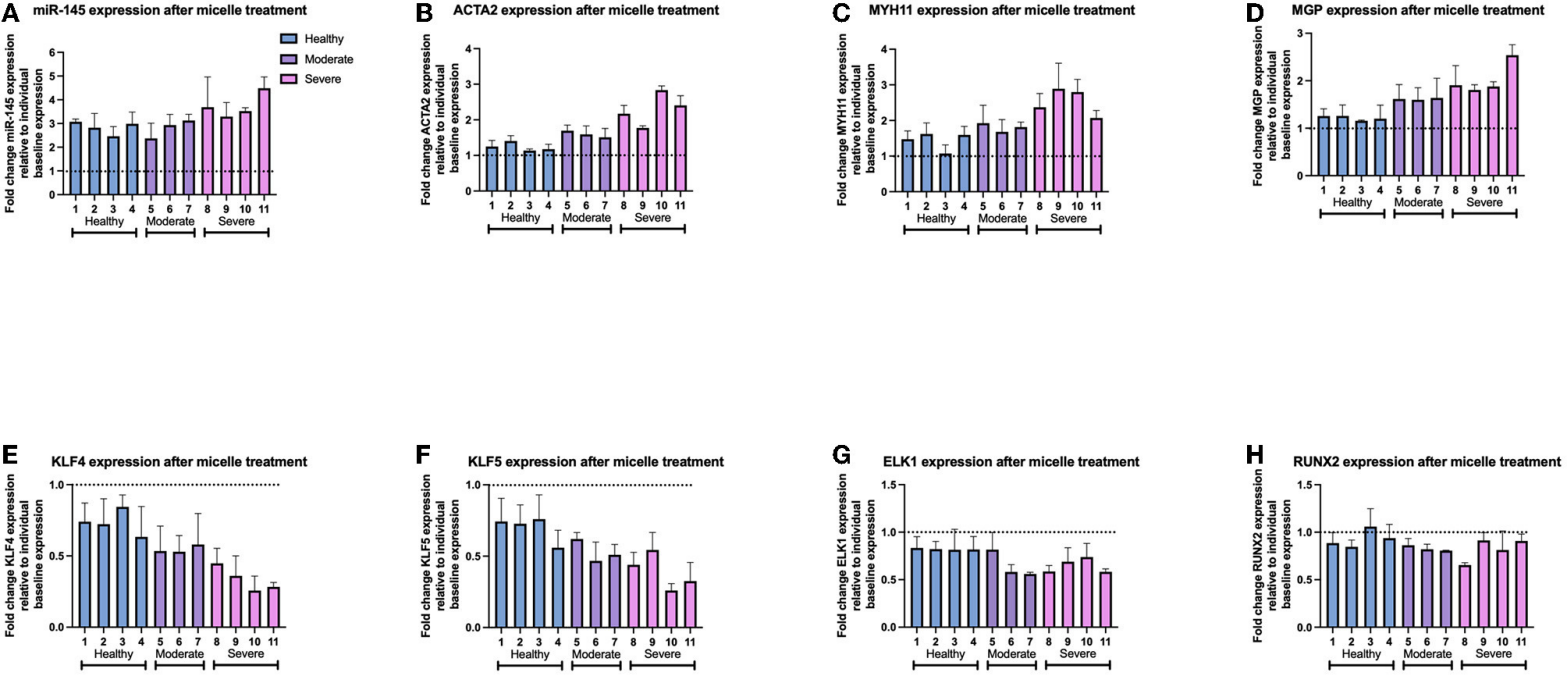
Gene expression of individual patient-derived VSMCs after miR-145 micelle therapy for **(A)** miR-145 levels, **(B)** ACTA2, **(C)** MYH11, **(D)** MGP, **(E)** KLF4, **(F)** KLF5, **(G)** ELK1, and **(H)** RUNX2. Dotted line represents individual sample baseline gene expression.

We then evaluated individual, patient-specific differential effects of miR-145 micelle treatment within disease groups. Analysis of individual sample responses to miR-145 micelle treatment reflected the trend toward a greater difference in gene expression based on disease severity shown in Figures 5, 6. In addition, a greater increase in miR-145 expression is accompanied with a greater decrease in KLF4 and KLF5 expression across individual samples within diseased groups. This is seen in sev-VSMC samples 10 and 11 which had a percent increase of miR-145 of 452.0 ± 14.2 and 428.6 ± 33.6%, respectively (Figure 6A). KLF4 and KLF5 expression relative to individual baselines were correspondingly decreased with expression levels of 25.71 ± 10.14 and 25.9 ± 4.82% for sample 10 and 28.31 ± 3.1 and 32.51 ± 13.80% for sample 11, respectively (Figures 6E,F). Thus, analysis of individual samples showed that with increased miR-145 expression, the direct targets of miR-145, KLF4/5, are downregulated for individual samples within disease groups. However, this did not directly translate to a proportional increase in contractile markers for individual samples. Nonetheless, miR-145 micelle treatment, on average, increases expression of contractile markers in all disease groups.

## Discussion

Current therapies for atherosclerosis are focused on prevention through lifestyle changes, LDL cholesterol management *via* statin therapy, endovascular therapies, and/or surgical intervention (31–33). However, at the time of statin prescription, many patients have already developed extensive atherosclerotic lesions and present vessel stenosis (34). Notably, Akyea et al. previously reported that aggressive statin therapy only marginally reduces risk of adverse cardiac events in an open population cohort studies of 183,213 participants (35). Additionally, certain subsets of patients demonstrate statin-intolerance due to adverse side effects (36). Thus, additional therapeutic strategies against atherosclerosis are needed.

In this study, we demonstrate the therapeutic potential of miR-145 micelles to restore healthy, contractile VSMC phenotype in atherogenic VSMCs derived from patient vascular tissue. miR-145 is a potent atheroprotective post-transcriptional regulator endogenously expressed in healthy VSMCs and downregulated in plaques and diseased VSMCs (12, 21, 22, 37). Accordingly, our results indicated that VSMCs derived from diseased arteries had decreased expression of miR-145 and contractile markers (ACTA2 and MYH11) relative to the healthy samples (Figures 1–3). However, treatment of the patient-derived VSMCs with mir-145 micelles resulted in the restoration of contractile markers to baseline healthy VSMC levels in diseased VSMCs (Supplementary Figure S4A–C). Hel-VSMCs also responded to miR-145 micelle by decreasing expression of synthetic markers KLF4 and KLF5 indicating a potential atheroprotective benefit by preventing healthy VSMCs from transitioning to a synthetic phenotype (Supplementary Figure S4E,F).

While miR-145 micelles inhibited expression of synthetic markers in VSMCs from varying disease states, therapeutic efficacy of the micelles scaled proportionally with disease severity (Figures 4, 5). The difference in response may be due to the miR to target site ratio as there are more binding sites for miR-145 in the sev-VSMCs compared to the hel-VSMCs, thus eliciting a greater modulatory effect (Figures 2E,F) (38–41). A similar pattern is observed for mod-VSMCs. Furthermore, as KLF4 inhibits expression of ACTA2 and MYH11, a greater decrease in KLF4 leads to a proportionally greater increase in expression of ACTA2 and MYH11, which is seen in sev-VSMCs compared to mod- or hel-VSMCs (Figures 4B,C) (42, 43).

Although we demonstrate the effects of miR-145 micelles on varying disease backgrounds and toward clinical application as an atherosclerosis therapy, we note a few limitations of this study. Firstly, our studies were limited in sample size due to the difficulty in obtaining routinely discarded patient tissue from vascular surgery. Further expansion of the patient VSMC sample bank will allow for greater number of moderately and severely diseased samples to delineate clearer differences in baseline gene expression and responses to miR-145 micelle treatment. Secondly, vessel disease severity and tissue viability were classified by the physician at the time of surgery and upon examination of the tissue. As this is a qualitative approach to categorizing patients and tissue, future work will utilize a more quantitative approach such as a coronary artery calcium score (CAC) or blood analysis for elevated LDL-cholesterol levels to categorize patient disease state (44). Nonetheless, our data demonstrates that miR-145 micelle treatment has the potential to abrogate atherosclerotic progression in patients with already significant atherosclerosis by promoting an atheroprotective VSMC phenotype in healthy and moderately diseased tissues.

## Conclusion

In summary, we collected patient-derived VSMCs from healthy, moderate, or severely diseased atherosclerotic arteries. Isolated VSMCs were characterized by immunocytochemistry and qRT-PCR for contractile and synthetic VSMC markers, and subsequently treated with miR-145 micelles to evaluate therapeutic response. Initial characterization of VSMCs showed that with increasing tissue disease severity there was decreased expression of miR-145 and contractile markers, however miR-145 micelle treatment restored expression of contractile markers in all diseased groups to baseline healthy levels. Interestingly, miR-145 micelle treatment had a differential effect with increasingly diseased VSMCs having the greatest therapeutic response to miR-145. Thus, we report a novel therapeutic which can potentially be used as an anti-atherosclerotic therapy during all stages of atherosclerosis diseases progression.

## Data Availability Statement

The original contributions presented in the study are included in the article/Supplementary Material, further inquiries can be directed to the corresponding author.

## Ethics Statement

The studies involving human participants were reviewed and approved by University of Southern California Institutional Review Board. The patients/participants provided their written informed consent to participate in this study.

## Author Contributions

EC conceived the project. EC, DC, and NP designed the experiments and wrote the manuscript. DC and NP performed the experiments and analyzed the data. All authors wrote the manuscript and have read and approved the final manuscript.

## Funding

The authors would like to acknowledge the financial support from University of Southern California, the American Heart Association Predoctoral Fellowship (19PRE34380998) awarded to DC, the National Science Foundation Graduate Student Fellowship awarded to NP, and Women in Science and Engineering (WiSE) and NIH New Innovator Award (DP2-DK121328) awarded to EC.

## Conflict of Interest

The authors declare that the research was conducted in the absence of any commercial or financial relationships that could be construed as a potential conflict of interest.

## Publisher's Note

All claims expressed in this article are solely those of the authors and do not necessarily represent those of their affiliated organizations, or those of the publisher, the editors and the reviewers. Any product that may be evaluated in this article, or claim that may be made by its manufacturer, is not guaranteed or endorsed by the publisher.
