# Supplementary material for: Therapeutic Response of miR-145 Micelles on Patient-Derived Vascular Smooth Muscle Cells
Source: Front Digit Health. 2022 Jun 15;4:836579. doi: 10.3389/fdgth.2022.836579 (PMC9240309; doi:10.3389/fdgth.2022.836579)
Supplement: Supplementary file 1 [file Data_Sheet_1.docx]

Supplemental Information

**Therapeutic response of miR-145 micelles on patient-derived vascular smooth muscle cells**

Neil Patel^a^, Deborah D. Chin^a^, Gregory A. Magee^b^, Eun Ji Chung*^a,b,c,d,e,f^

^a^Department of Biomedical Engineering, University of Southern California, Los Angeles, CA, 90089, United States

^b^Division of Vascular Surgery and Endovascular Therapy, Department of Surgery, Keck School of Medicine, University of Southern California, Los Angeles, CA, 90033, United States

^c^Mork Family Department of Chemical Engineering and Materials Science, University of Southern California, Los Angeles, CA, 90089, United States

^d^Eli and Edythe Broad Center for Regenerative Medicine and Stem Cell Research, Keck School of Medicine, University of Southern California, Los Angeles, CA, 90033, United States

^e^Division of Nephrology and Hypertension, Department of Medicine, Keck School of Medicine, University of Southern California, Los Angeles, CA, 90033, United States

^f^Norris Comprehensive Cancer Center, Keck School of Medicine, University of Southern California, Los Angeles, CA, 90089, United States

*Corresponding author

Eun Ji Chung

Department of Biomedical Engineering

University of Southern California

1042 Downey Way, DRB 140

Los Angeles, CA 90089

Tel.: +1-213-740-2925

Fax: +1-213-821-3897

Email: eunchung@usc.edu

**Keywords**

Nanomedicine, atherosclerosis, microRNA-145, personalized medicine, vascular smooth muscle cells, plaque


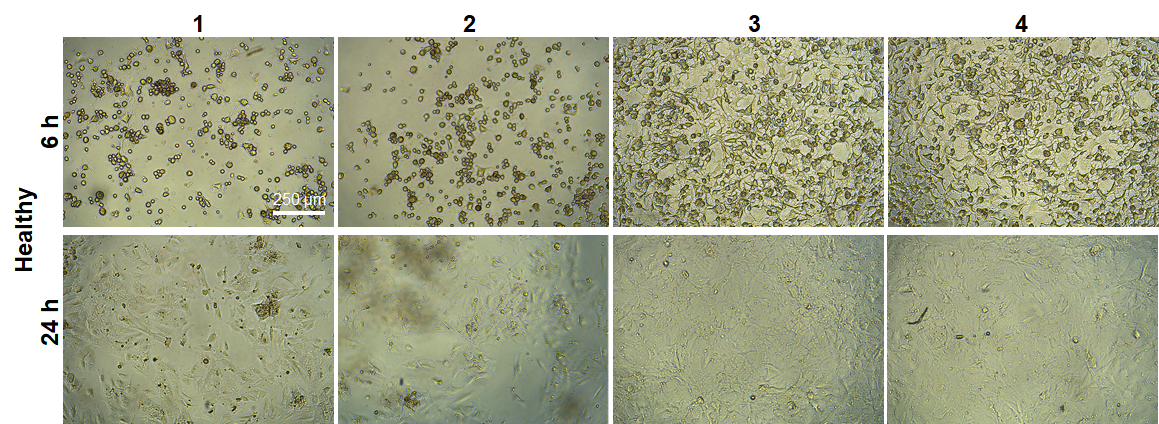

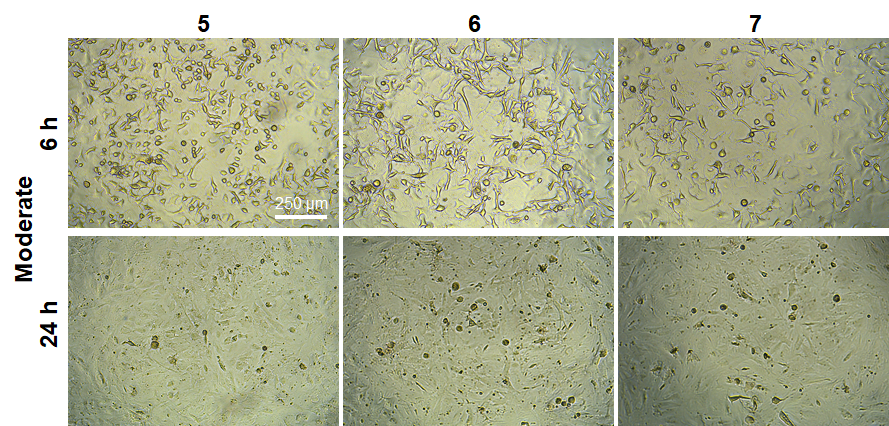

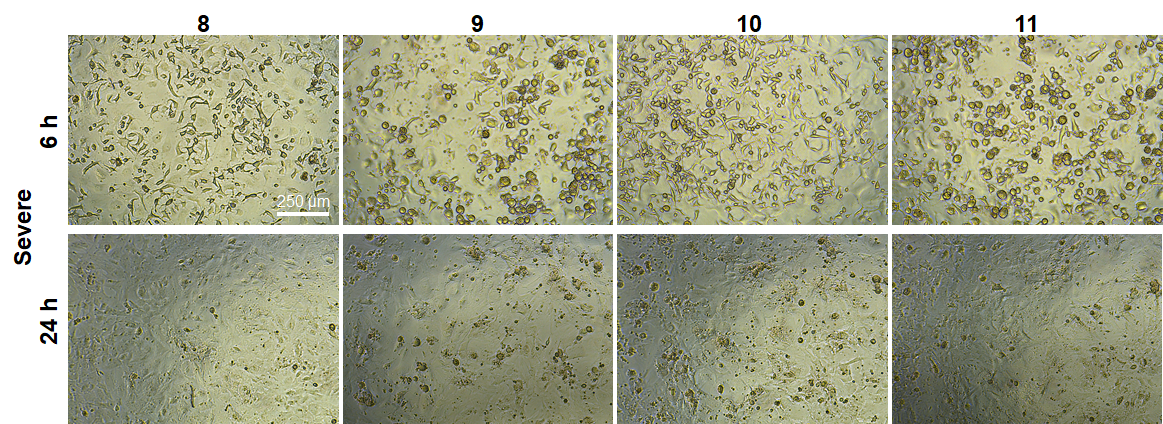


**Figure S1.** Bright field images of patient-derived VSMCs 6 hours after seeding. While some samples (1, 2, 9, and 11) took longer to adhere, no significant differences in morphology between healthy (1-4), moderate (5-7), and severe (8-11) disease VSMCs were found 24 hours after seeding. Scale bar 250 µm.

**Figure S2**. Quantification of ACTA2 (circles) and CD68 (squares) expression from immunocytochemistry images of healthy, moderate, and severely diseased samples.


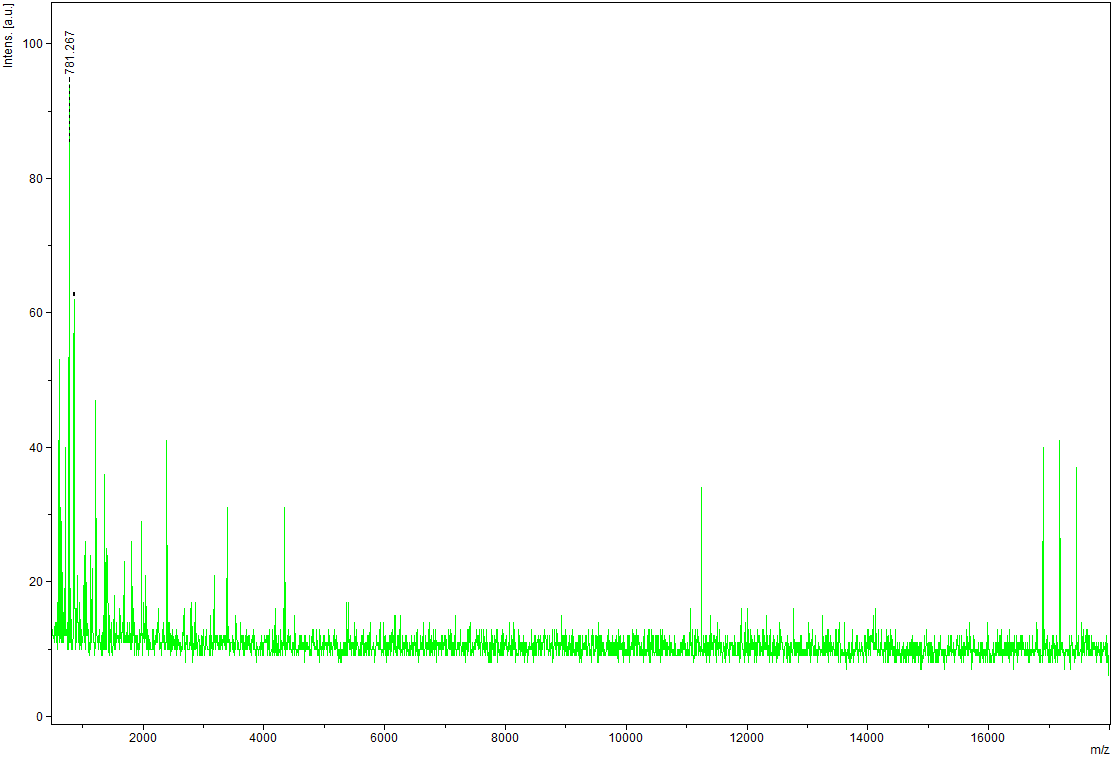


17,265 m/z

**Figure S3.** MALDI-TOF mass spectrum of DSPE-PEG(2000)-miR-145 at 17265 m/z (Expected: 17047 Da), confirming successful synthesis of DSPE-PEG(2000)-miR-145 peptide amphiphile.


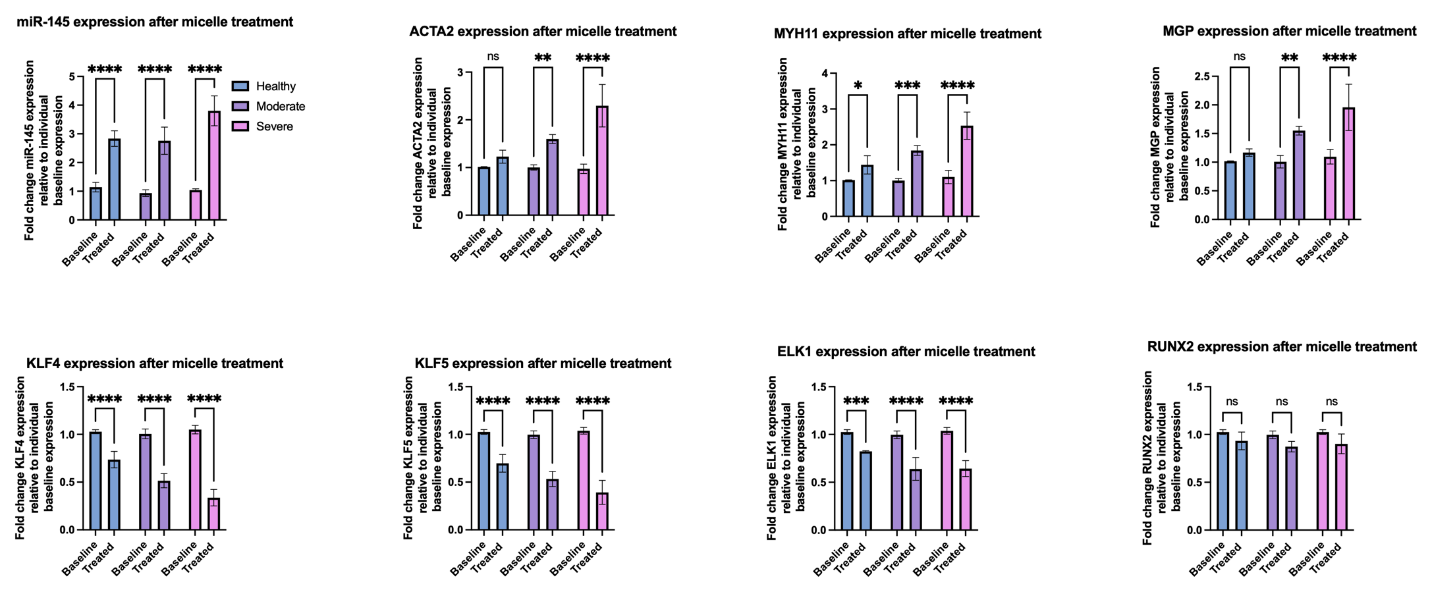


**Figure S4.** Gene expression of VSMCs after miR-145 micelle treatment relative to baseline expression for each group. A) miR-145 levels, B) ACTA2, C) MYH11, D) MGP, E) KLF4, F) KLF5, G) ELK1, and H) RUNX2. *indicates p<0.05, ** p<0.01, *** p<0.001, and ****p<0.0001, ns not significant.

**Figure S5.** Gene expression of VSMCs after miR-145 micelle treatment relative to the average baseline expression of healthy VSMCs (dotted line) for atheroprotective markers (miR-145, ACTA2, MYH11, MGP) and atherogenic markers (KLF4, KLF5, ELK1, RUNX2).


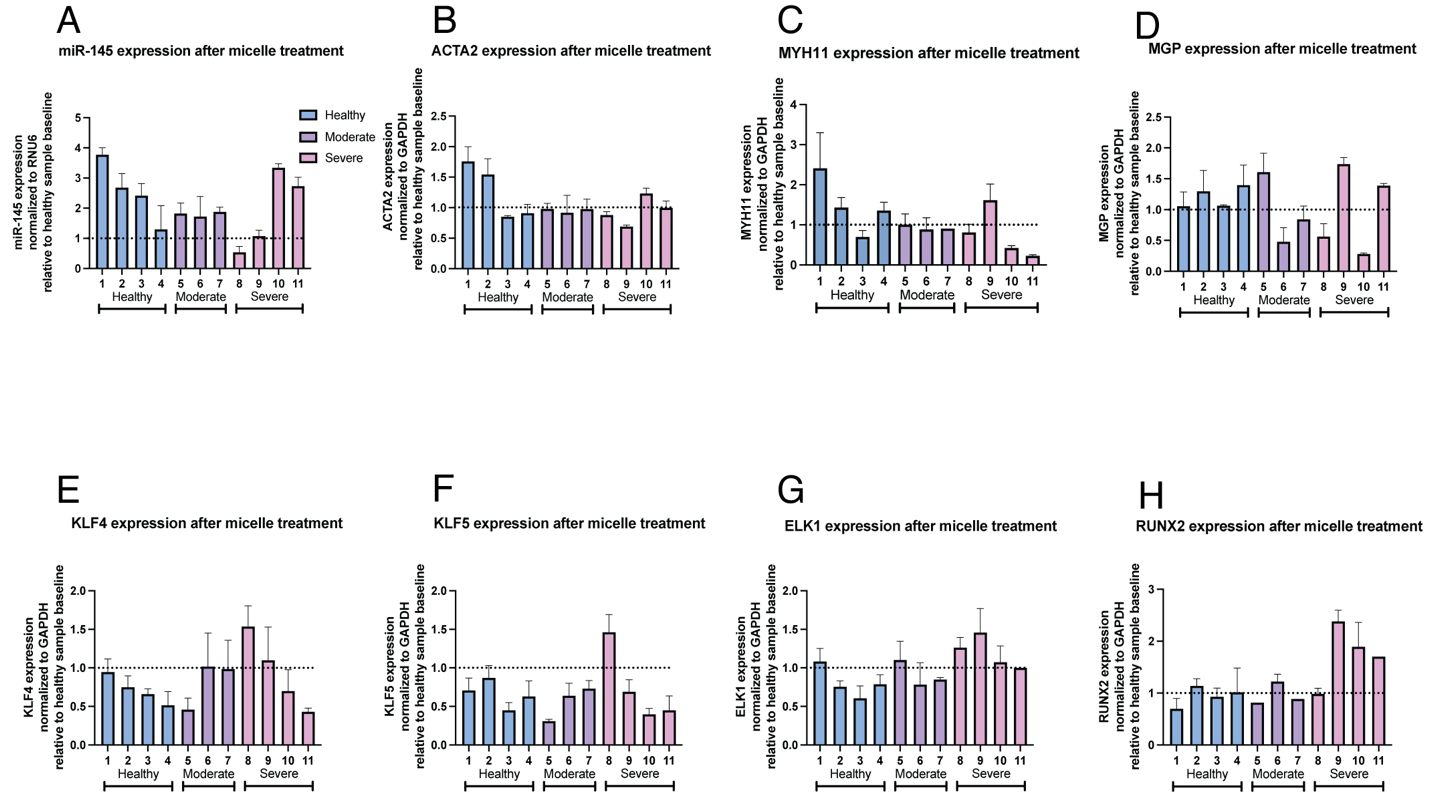


**Figure S6**. Gene expression of individual VSMCs after miR-145 micelle treatment relative to healthy VSMC baseline expression (dotted line) for A) miR-145 levels, B) ACTA2, C) MYH11, D) MGP, E) KLF4, F) KLF5, G) ELK1, and H) RUNX2.
